# Supplementary material for: High Throughput Manufacturing of Bio-Resorbable Micro-Porous Scaffolds Made of Poly(L-lactide-co-ε-caprolactone) by Micro-Extrusion for Soft Tissue Engineering Applications
Source: Polymers (Basel). 2019 Dec 24;12(1):34. doi: 10.3390/polym12010034 (PMC7023538; doi:10.3390/polym12010034)
Supplement: Supplementary file 1 [file polymers-12-00034-s001.pdf]

polymers

MDPI

Supplementary material:

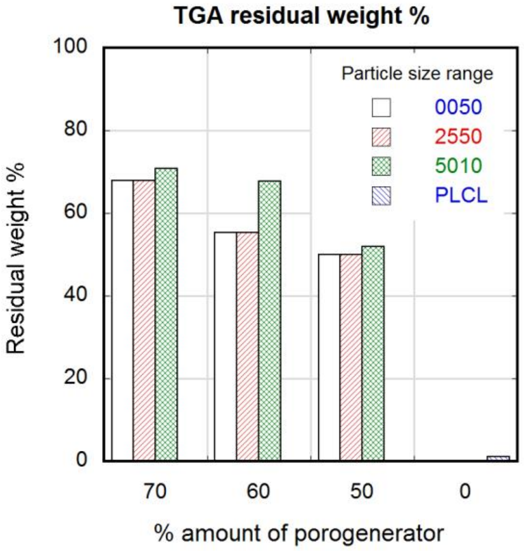

Figure S1. Percental residual weight measured by TGA for samples containing different particle sizes (white 0–50 μm, upwards stripe pattern 25–50 μm, cross pattern 50–100 μm, downwards strip pattern neat PLCL) as a function of the theoretical percental amount of porogenerator.

$$\varphi_v = \frac{\rho_m}{\rho_p} \cdot \frac{\varphi_w}{1 + \varphi_w \left(\frac{\rho_m}{\rho_p} - 1 \right)}$$

Equation 1. Equation used to calculate the volume fraction (φ_v) function of matrix density (ρ_m), particle density (ρ_p) and weight fraction (φ_w).

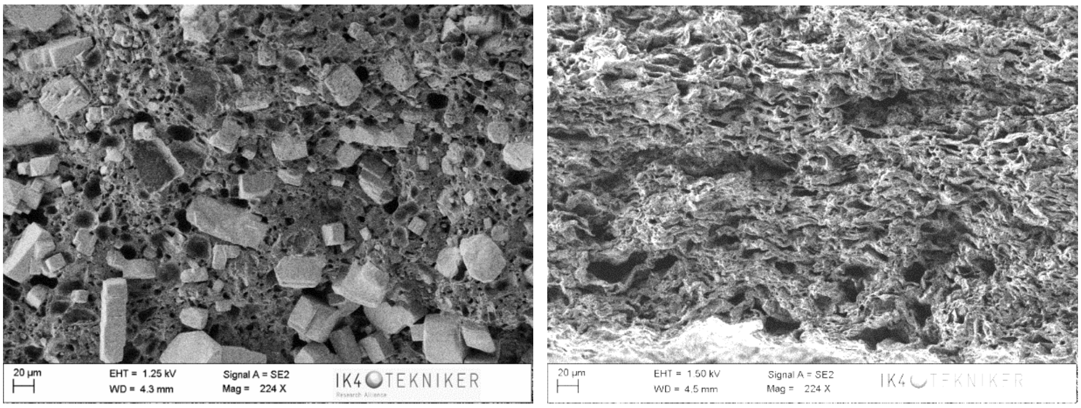

Figure S2. Sample 005050 containing 50 wt.% of salt in 0–50 μm range; non leached (left), leached (right).

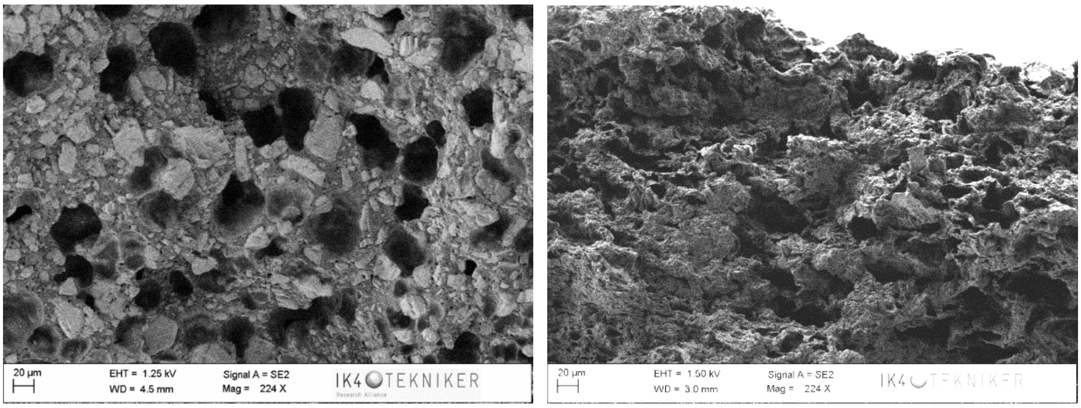

Figure S3. Sample 005060 containing 60 wt.% of salt in 0–50 µm range; non leached (left), leached (right).

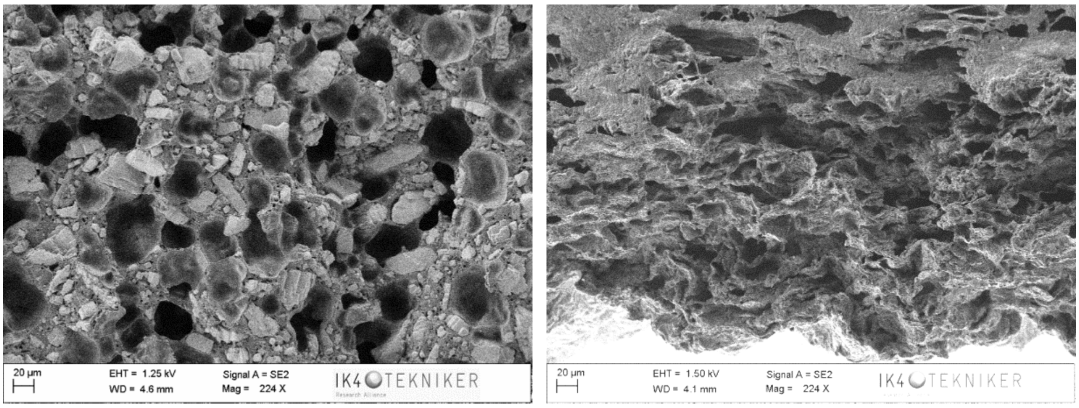

Figure S4. Sample 005070 containing 70 wt.% of salt in 0–50 µm range; non leached (left), leached (right).

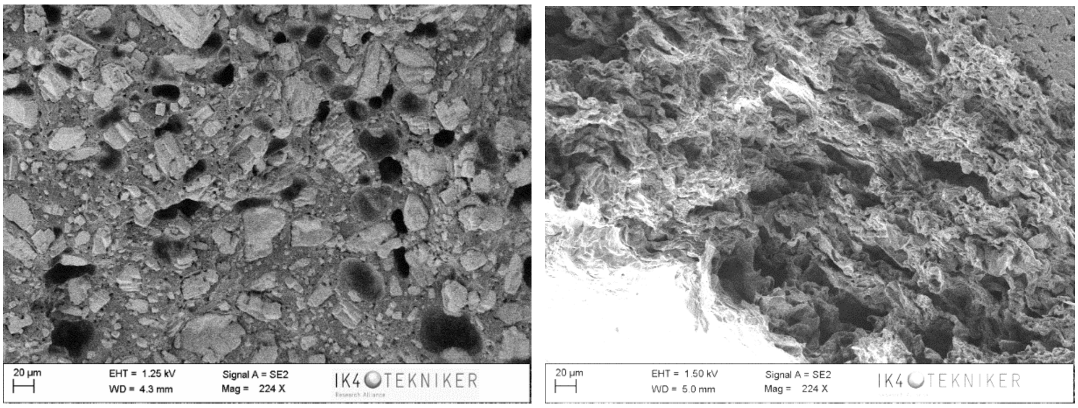

Figure S5. Sample 255050 containing 50 wt.% salt in 25–50 µm range; non leached (left), leached (right).

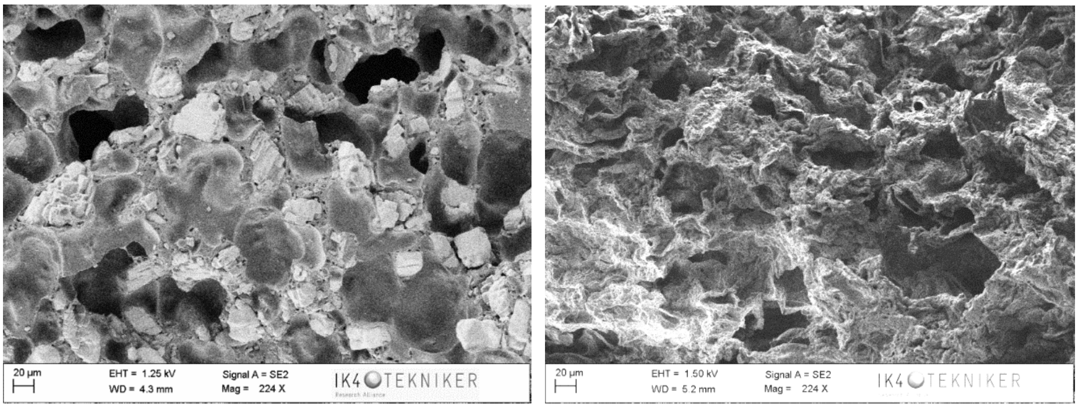

Figure S6. Sample 255060 containing 60 wt.% of salt in 25–50 range; non leached (left), leached (right).

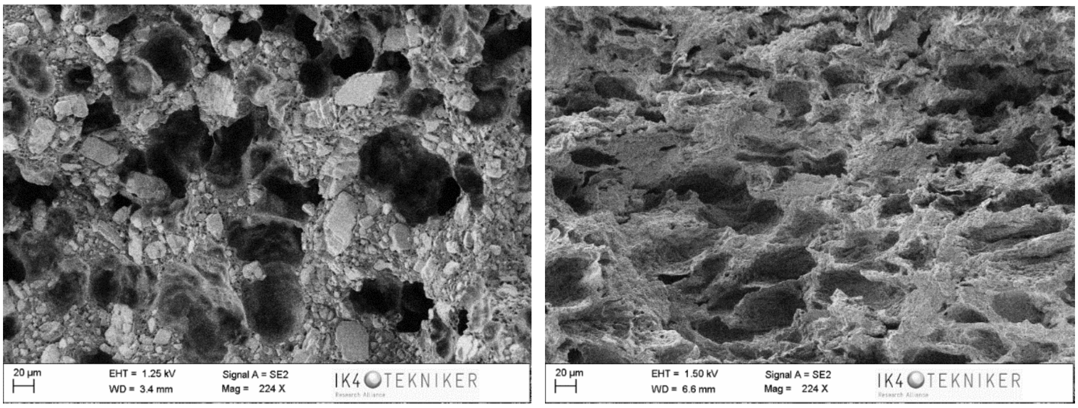

Figure S7. Sample 255070 containing 70 wt.% of salt in 25–50 µm range; non leached (left), leached (right).

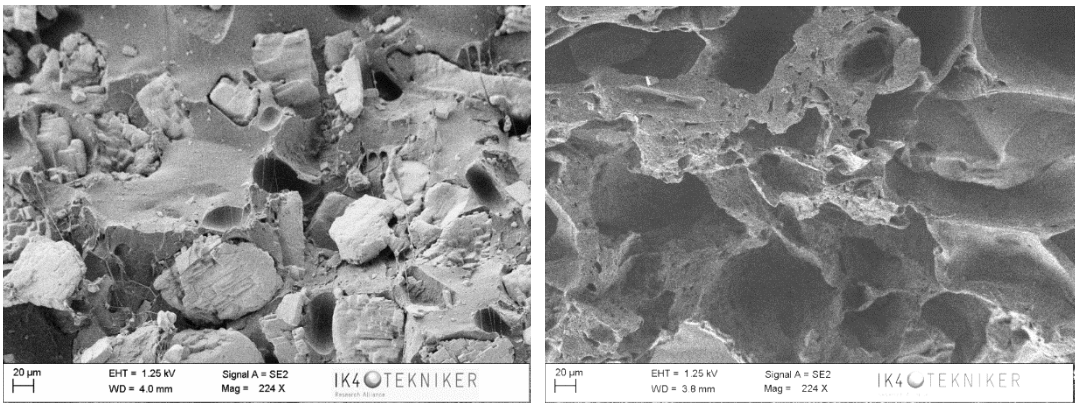

Figure S8. Sample 501050 containing 50 wt.% of salt in 50–100 µm range; non leached (left), leached (right).

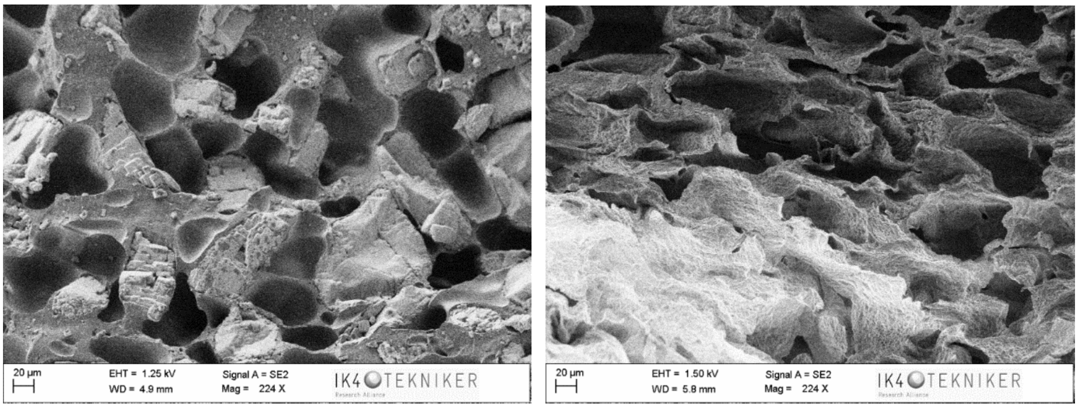

Figure S9. Sample 501060 containing 60 wt.% of salt in 50–100 µm range; non leached (left), leached (right).

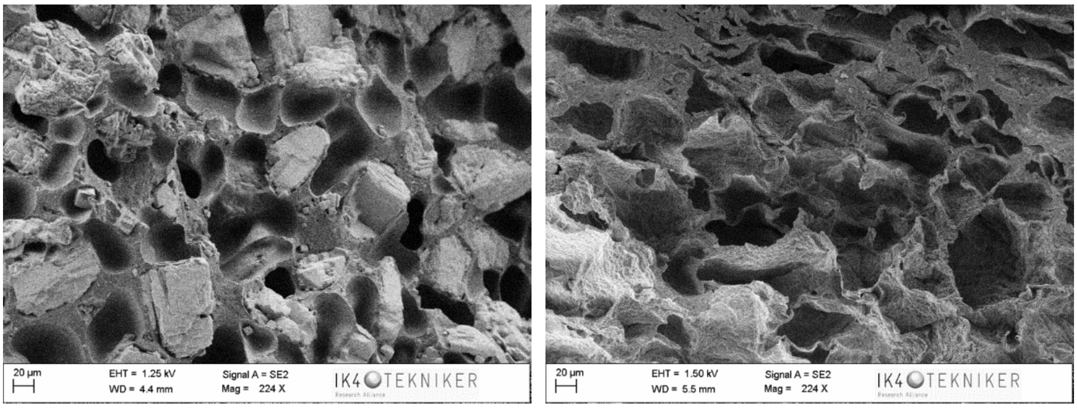

Figure S10. Sample 501070 containing 70 wt.% salt in 50–100 µm range; non leached (left), leached (right).

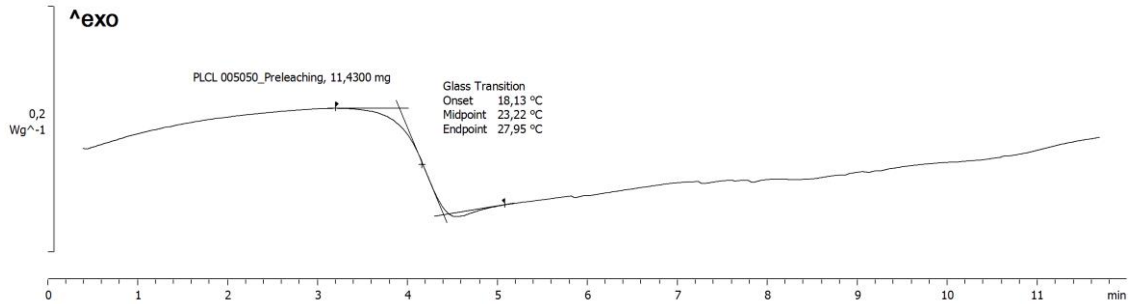

Figure S11. DSC curve of non leached sample 005050 containing 50 wt.% salt in 0–50 µm range, exothermic upwards.

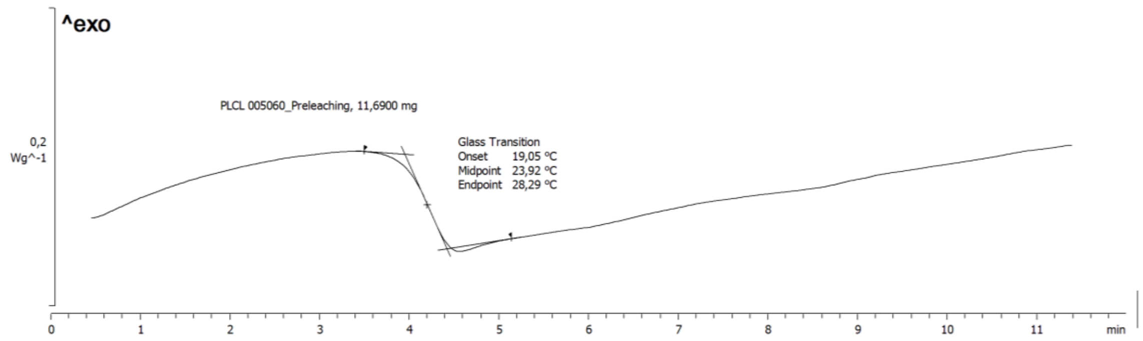

Figure S12. DSC curve of non leached sample 005060 containing 60 wt.% salt in 0–50 μm range, exothermic upwards.

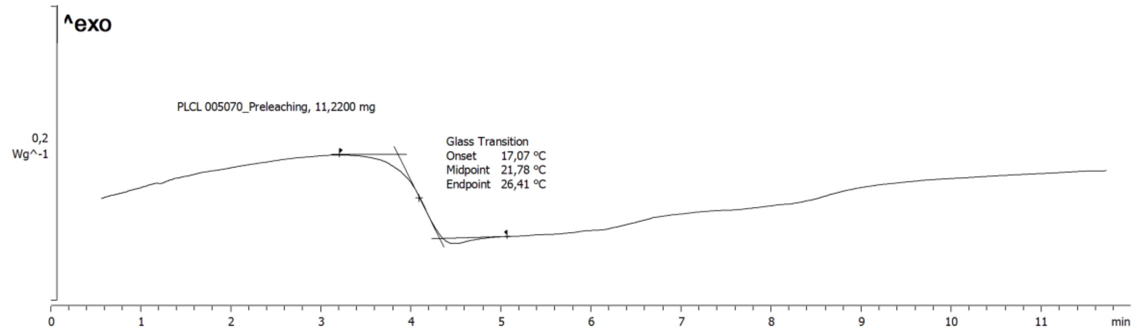

Figure S13. DSC curve of non leached sample 005070 containing 70 wt.% salt in 0–50 μm range, exothermic upwards.

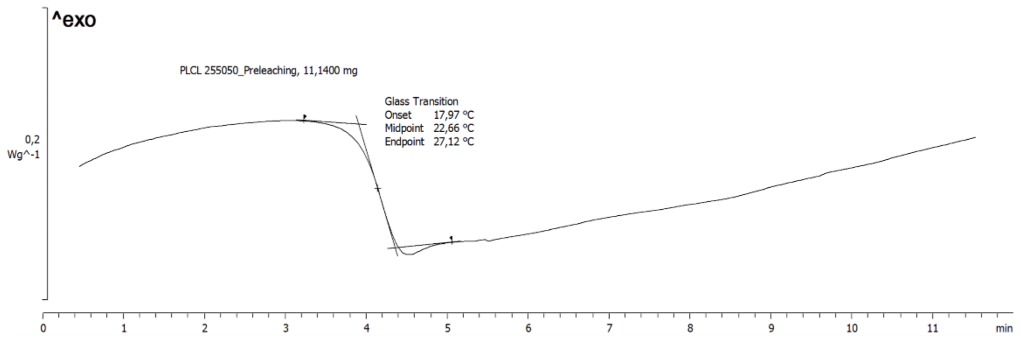

Figure S14. DSC curve of non leached sample 255050 containing 50 wt.% salt in 25–50 μm range, exothermic upwards.

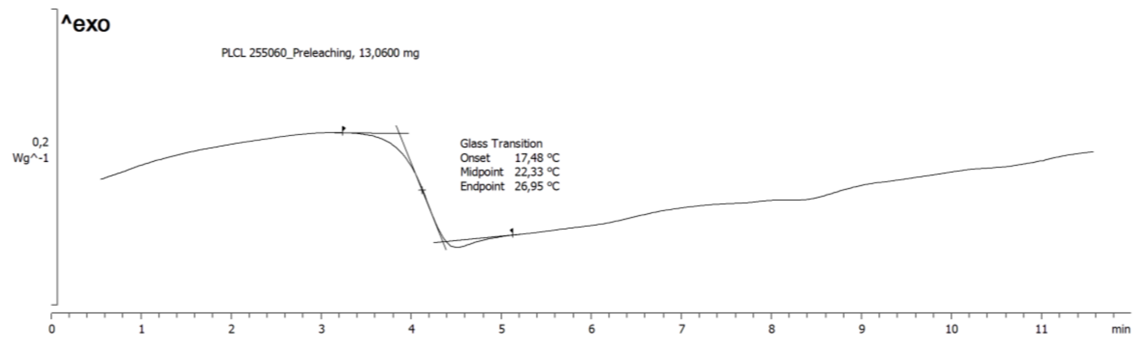

Figure S15. DSC curve of non leached sample 255060 containing 60 wt.% salt in 25–50 μm range, exothermic upwards.

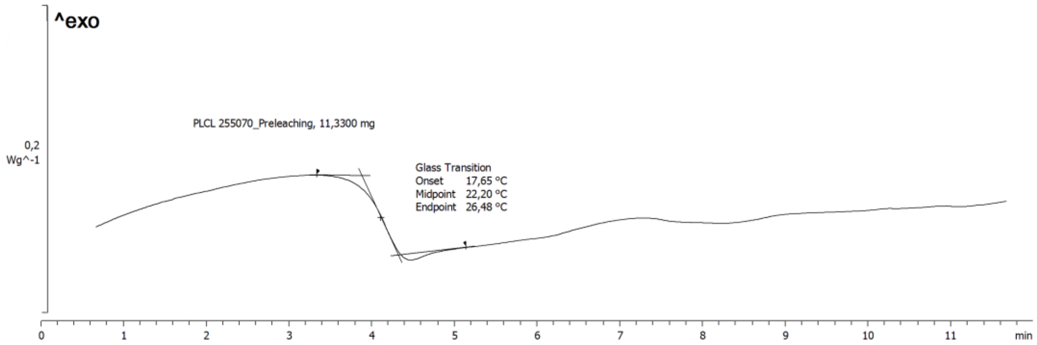

Figure S16. DSC curve of non leached sample 255070 containing 70 wt.% salt in 25–50 μm range, exothermic upwards.

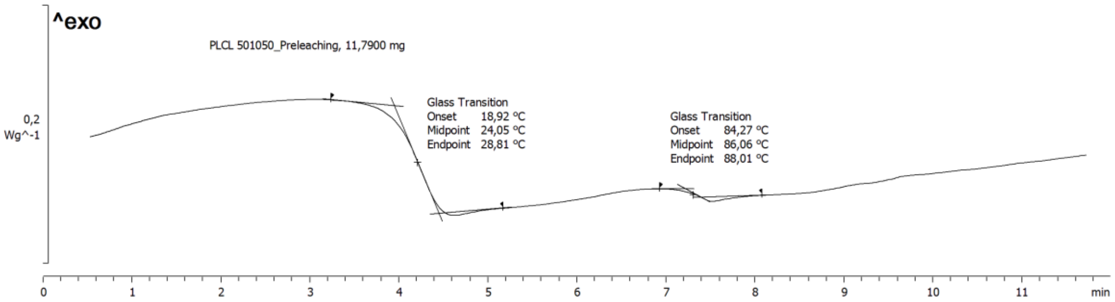

Figure S17. DSC curve of non leached sample 501050 containing 50 wt.% salt in 50–100 μm range, exothermic upwards.

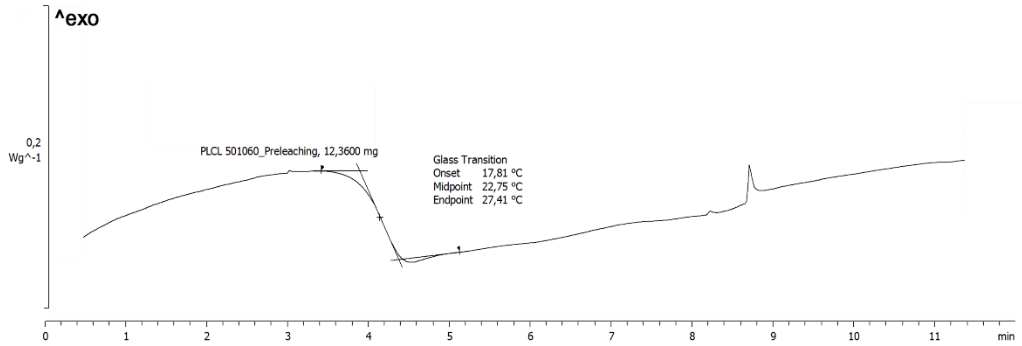

Figure S18. DSC curve of non leached sample 501060 containing 60 wt.% salt in 50–100 μm range, exothermic upwards.

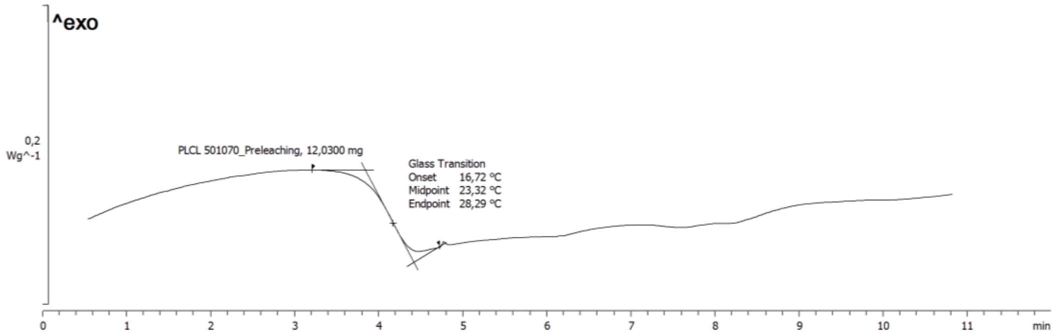

Figure S19. DSC curve of non leached sample 501070 containing 70 wt.% salt in 50–100 μm range, exothermic upwards.

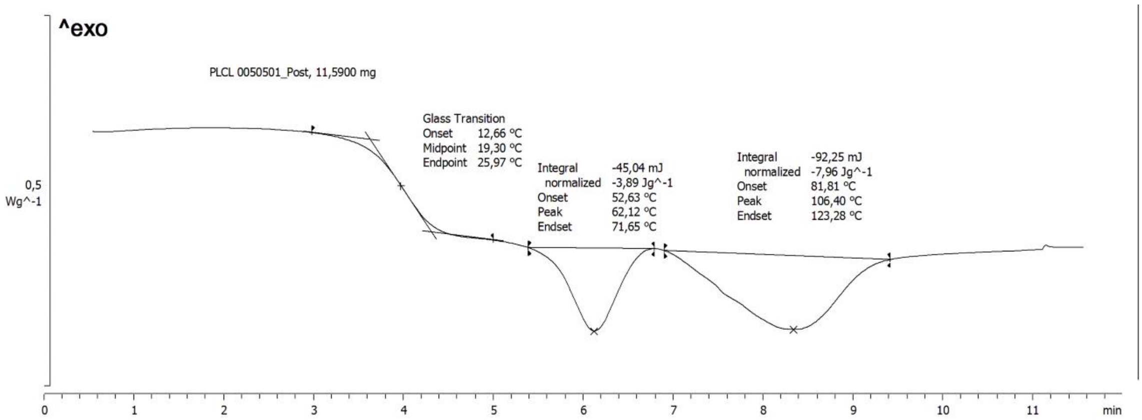

Figure S20. DSC curve of leached sample 005050 containing 50 wt.% salt in 0–50 μm range, exothermic upwards.

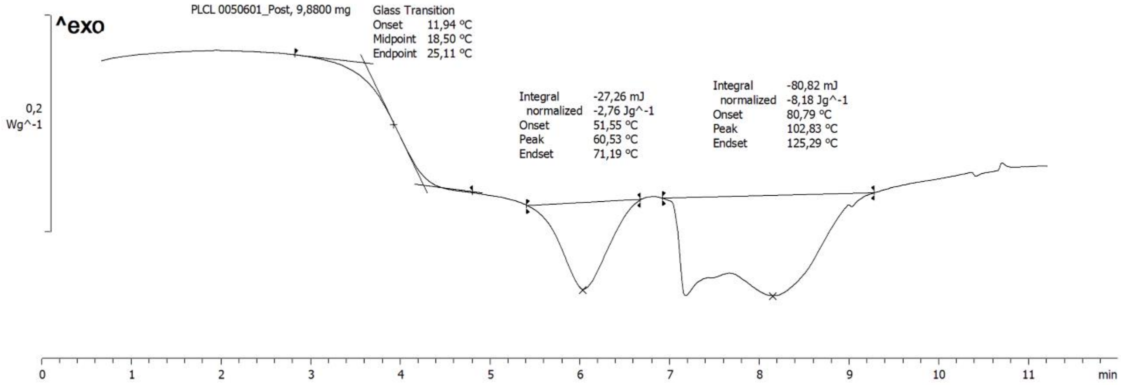

Figure S21. DSC curve of leached sample 005060 containing 60 wt.% salt in 0–50 μm range, exothermic upwards.

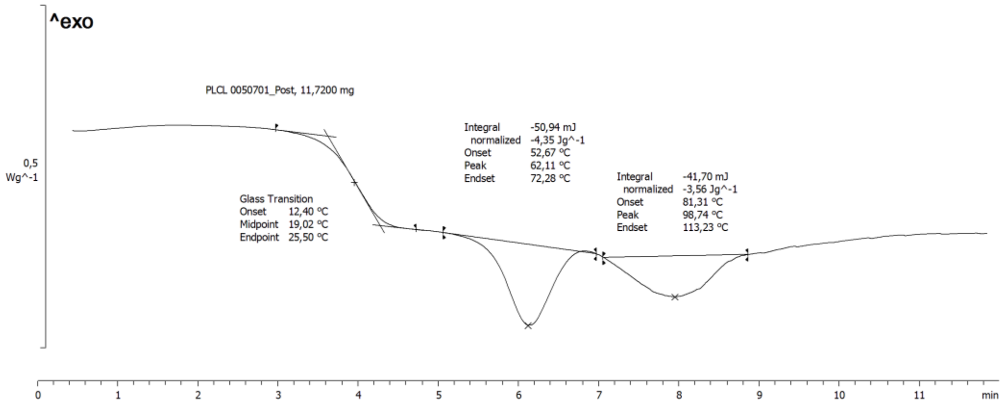

Figure S22. DSC curve of leached sample 005070 containing 70 wt.% salt in 0–50 μm range, exothermic upwards.

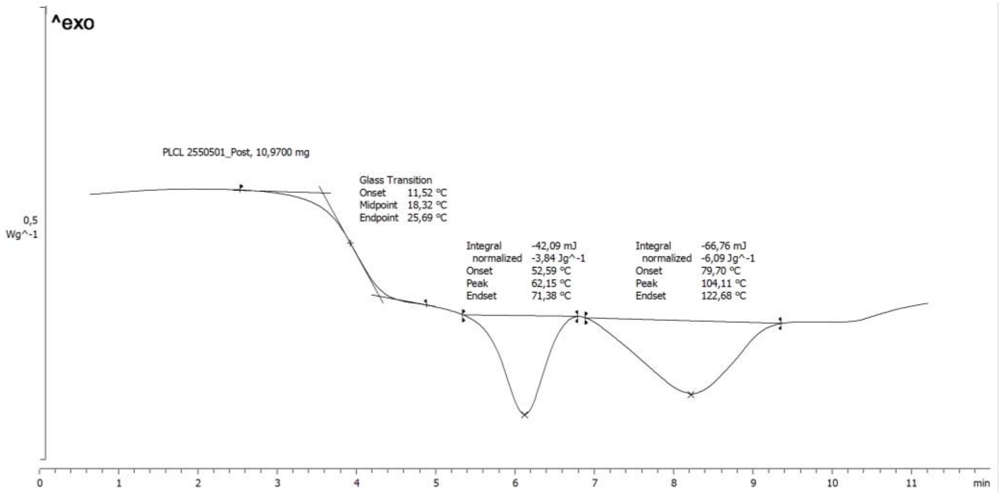

Figure S23. DSC curve of leached sample 255050 containing 50 wt.% salt in 25–50 μm range, exothermic upwards.

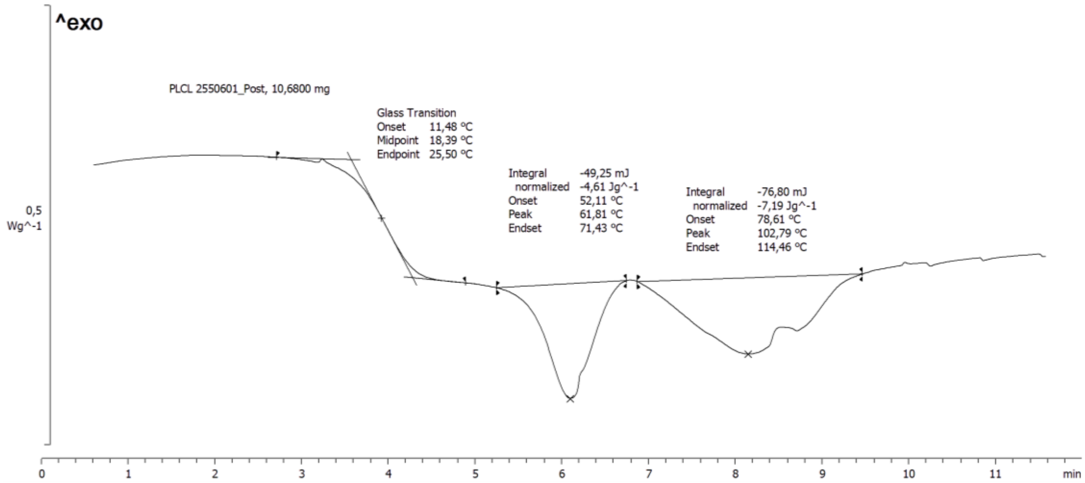

Figure S24. DSC curve of leached sample 255060 containing 60 wt.% salt in 25–50 μm range, exothermic upwards.

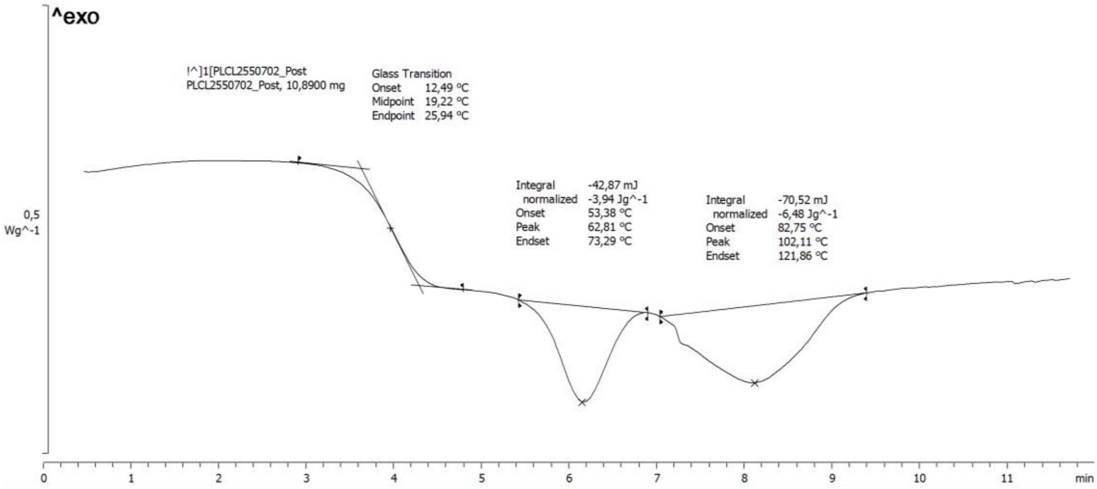

Figure S25. DSC curve of leached sample 255070 containing 70 wt.% salt in 25–50 μm range, exothermic upwards.

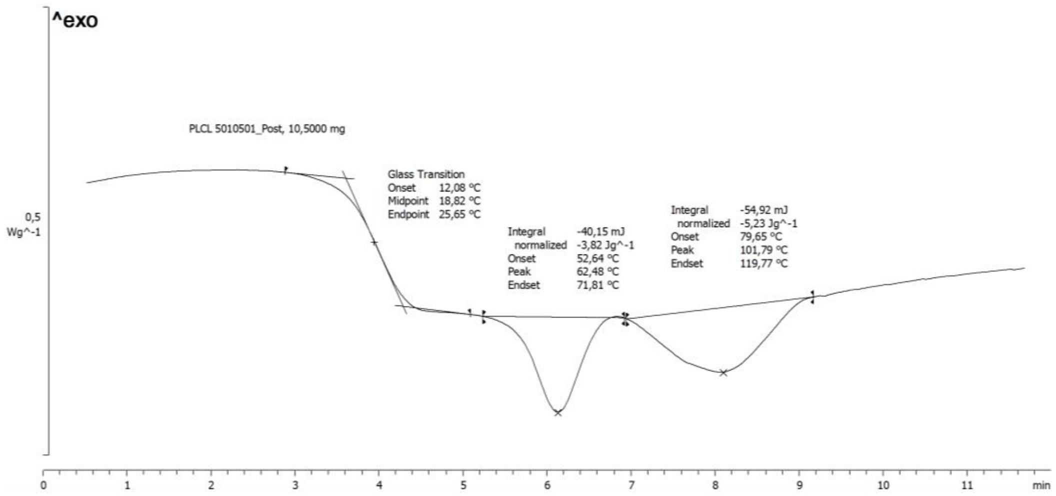

Figure S26. DSC curve of leached sample 501050 containing 50 wt.% salt in 50–100 µm range, exothermic upwards.

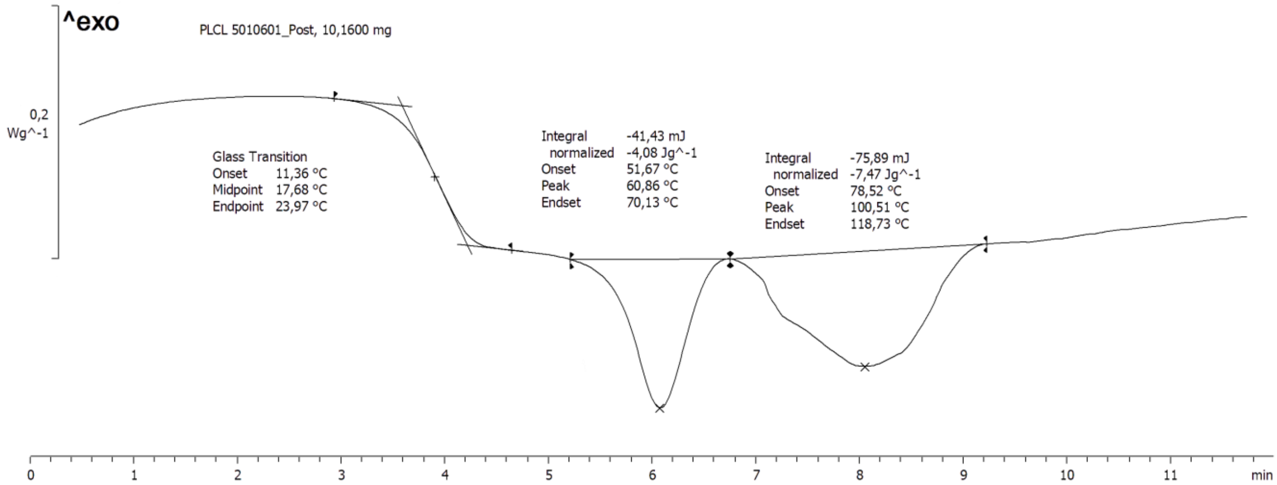

Figure S27. DSC curve of leached sample 501060 containing 60 wt.% salt in 50–100 µm range, exothermic upwards.

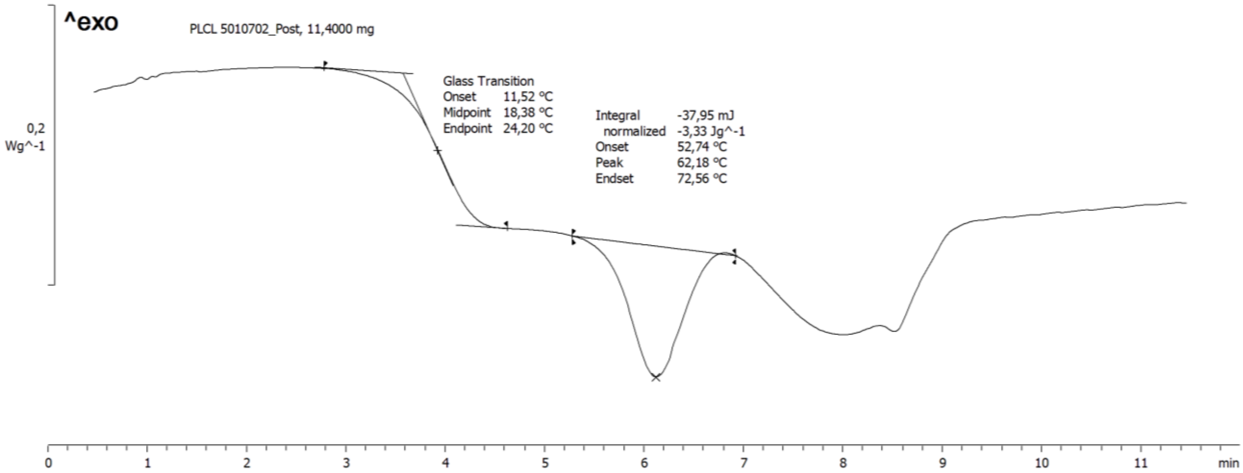

Figure S28. DSC curve of leached sample 501070 containing 70 wt.% salt in 50–100 µm range, exothermic upwards.

© 2019 by the authors. Submitted for possible open access publication under the terms and conditions of the Creative Commons Attribution (CC BY) license (http://creativecommons.org/licenses/by/4.0/).
